# Effect of a dual orexin receptor antagonist on Alzheimer's disease: Sleep disorders and cognition

**DOI:** 10.3389/fmed.2022.984227

**Published:** 2023-02-01

**Authors:** Mengzhen Zhou, Shi Tang

**Affiliations:** ^1^Department of Neurology, The First Affiliated Hospital of Shandong First Medical University & Shandong Provincial Qianfoshan Hospital, Jinan, Shandong, China; ^2^Department of Neurology, Shandong Provincial Hospital Affiliated to Shandong First Medical University, Jinan, Shandong, China

**Keywords:** Alzheimer's disease, cognition, insomnia, orexin, treatment

## Abstract

Orexin is a neuropeptide produced by the lateral hypothalamus that plays an important role in regulating the sleep-wake cycle. The overexpression of the orexinergic system may be related to the pathology of sleep/wakefulness disorders in Alzheimer's disease (AD). In AD patients, the increase in cerebrospinal fluid orexin levels is associated with parallel sleep deterioration. Dual orexin receptor antagonist (DORA) can not only treat the sleep-wakefulness disorder of AD but also improve the performance of patients with cognitive behavior disorder. It is critical to clarify the role of the orexin system in AD, study its relationship with cognitive decline in AD, and evaluate the safety and efficacy of DORA.

## 1. Introduction

Sleep can increase the clearance of neurotoxic products of neuronal activities accumulated in the awake brain (1). Long-term sleep disorders and insufficient sleep will cause one or more cognitive function impairments, affecting daily life and social ability (2). Studies have found that 25%−60% of Alzheimer's disease (AD) patients have sleep disorders, such as circadian rhythm disorder, fragmented sleep and reduced night sleep (3). The increase in orexin-A in patients with moderate to severe AD can be involved in the occurrence of sleep disorders (4, 5).

Orexin neurons, which originate from the lateral hypothalamus, widely project to brain regions related to arousal and cognition, such as the medial prefrontal cortex (mPFC), basal forebrain (BF) and hippocampus (Hip) (6). They play an important regulatory role in many physiological processes, such as feeding, energy metabolism, sleep/wakefulness and neuroendocrine homeostasis. Dual orexin receptor antagonist (DORA) improves sleep by inhibiting hyperactive arousal pathways caused by orexin signaling in insomnia patients. Meanwhile, learning and memory are regulated by stimulating neurogenesis in the dentate gyrus of the hippocampus (7).

**Figure 1:**
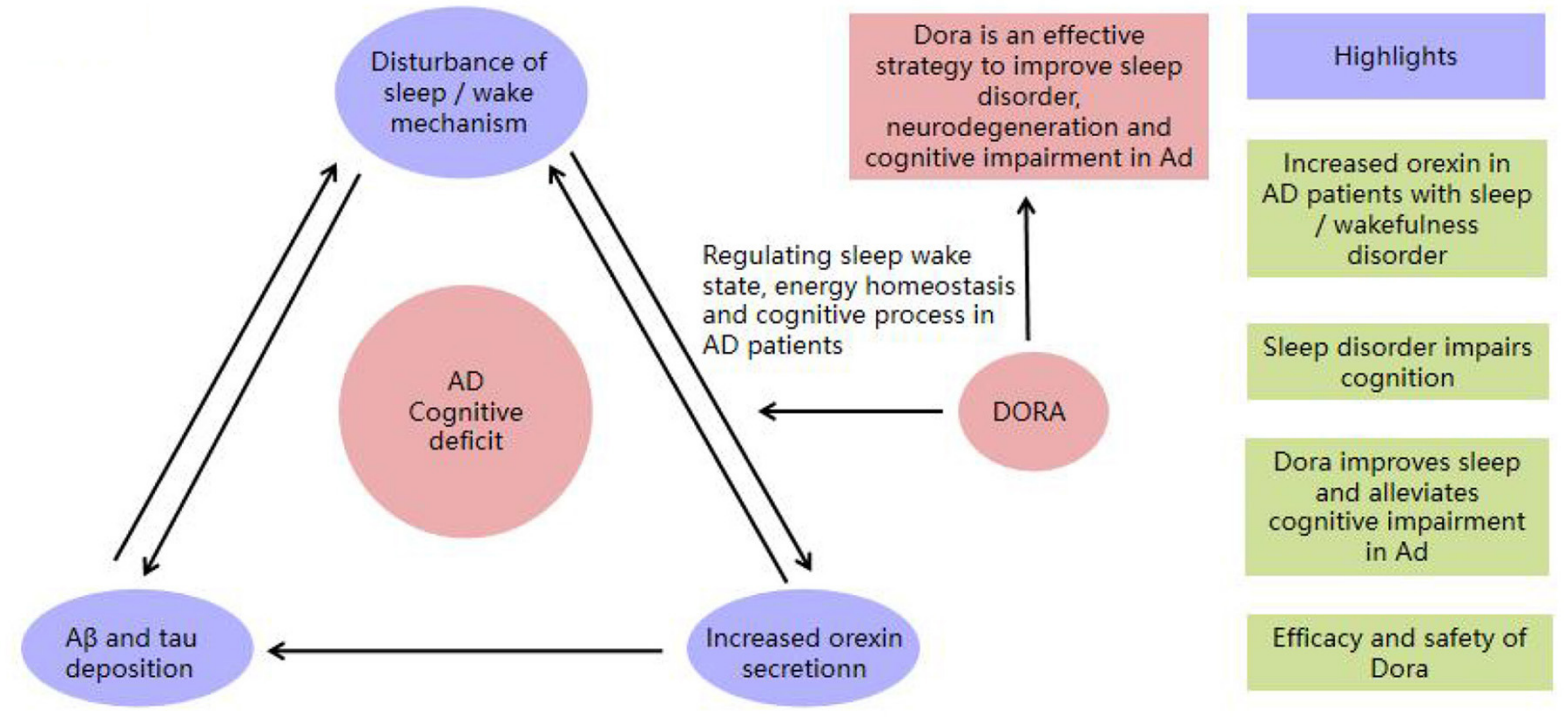
The schematic diagram of DORA intervention in AD.

Figure 1 shows a schematic diagram of DORA intervention in AD.

## 2. Increased orexins in AD patients with sleep/wakefulness disorder

### 2.1. Possible causes of orexin system disorder in AD

The cholinergic basal forebrain is one of the key structures that promotes the awakening system and plays a central role in cognitive dysfunction in AD (8, 9). The loss of cholinergic signals is thought to be closely related to the decline in attention and memory function (10). Neurons that promote awakening (11), especially basal forebrain cholinergic neurons, die during neurodegeneration in AD (12). This loss may lead to the upregulation of other arousal systems, such as the activity of orexin neurons, which is a compensatory mechanism involved in the lateral hypothalamus during AD neurodegeneration (13).

As a compensatory mechanism of cholinergic dysfunction, the impact of the overexpression of the orexin system on cognitive function is unknown in AD. The first possibility is that orexin system hyperactivity in AD patients is overexpressed to compensate for cholinergic dysfunction and maintain cognitive function to a certain extent. The second is that the hyperarousal caused by orexin overexpression may impair cognitive function (14, 15).

### 2.2. Interaction between sleep/wakefulness disorder and AD

Sleep**/**wakefulness disorders are common symptoms in AD patients, such as circadian rhythm disorder, fragmented sleep, insomnia and excessive daytime sleepiness (16). Among them, the striking problems are excessive awakening at night (23%), early awakening (11%) and excessive sleepiness during the day (10%) (17, 18). Sleep/wakefulness disorders are serious enough to interfere with normal physiological, psychological, social and emotional functions in patients with AD. The relationship between AD and sleep/wakefulness disorder is likely to be complex and bidirectional, and subjects with sleep/wakefulness disorder have a 1.49-fold increased risk of AD (12). Sleep/wakefulness disorders usually occur before cognitive decline.

The increase in orexin in the cerebrospinal fluid (CSF) of AD patients leads to an increase in wakefulness during the night, prolonged sleep latency (LPS), decreased sleep efficiency (SE) and rapid eye movement sleep (REM) (19). For each percentage reduction in REM sleep, the risk of dementia increases by 9%. Maintaining the normal orexinergic signal is considered to be the key to ensuring the circadian rhythm of the whole sleep-wake cycle (20). Therefore, whether DORA can improve sleep and delay the progression of AD has become a major hot issue in recent years (21).

### 2.3. The mechanism by which sleep/wakefulness disorder damages learning and memory in AD

Sleep/wakefulness behavior is a basic brain function that is closely related to cognition and synaptic plasticity and is essential to ensure brain metabolic homeostasis. Sleep can enlarge the interstitial space of the cerebral cortex (22), which increases convective exchange between cerebrospinal fluids and interstitial fluids. Sleep helps to clear the potential products from neurotoxic and neuroactive degradation accumulated in the awake brain (23).

The circadian rhythm is considered to be the key to hippocampal-dependent memory formation and consolidation, which regulates various physiological events, including cognitive performance and memory (24). Circadian rhythm disruption may damage the clearance of β-amyloid (Aβ) and the microtubule-associated protein tau in the brain glymphatic system (25), increase local brain oxidative stress, and reduce circulating melatonin levels, which ultimately leads to cognitive dysfunction and exacerbates the risk of AD (26, 27).

### 2.4. The bidirectional relationship between Aβ deposition, tau protein phosphorylation and sleep/wakefulness disorder in AD pathology

As a key protein in the pathogenesis of AD, Aβ is formed by abnormal aggregation in brain tissues, which leads to the occurrence and development of AD, known as the “amyloid cascade hypothesis” (28). At the same time, the neurofibrillary tangle caused by tau protein hyperphosphorylation is also an important feature of the pathological changes in AD. Sleep/wakefulness disorder leads to the abnormal phosphorylation of tau protein. Hyperphosphorylated tau protein reduces the binding force between tau protein and tubulin and then promotes the formation of double helix fibers. The twisting and twining of double helix filaments leads to tangles of nerve fibrils, which causes abnormal neuron structure and function and produces brain neurodegenerative disease (29, 30). Sleep/wakefulness disorder significantly increased Aβ levels and promoted the formation of senile plaques in AD mice (8). The relationship between sleep/wakefulness disorders and Aβ deposition may be bidirectional: sleep/wakefulness disorder causes Aβ deposition and further interrupts the sleep process (31).

## 3. Sleep/wakefulness disorder damages cognition

### 3.1. Sleep reactivates and consolidates newly acquired memories

Sleep is conducive to reactivating and consolidating newly acquired memories and making them more profound (32). Our brain selects noteworthy information for reprocessing during sleep. Researchers generally believe that both REM sleep and slow wave sleep (SWS) play an important role in memory processing. In addition to strengthening established memory, sleep also supports the transformation of memory between the hippocampus and the neocortex, integrating memory in the neocortex into a broader connection (33).

### 3.2. Sleep promotes information reorganization and active system integration

During sleep, the cerebral cortex integrates and processes short-term memory and long-term memory to form new synaptic connections, which is conducive to the effective recovery of energy and the improvement of energy for the brain (34, 35).

Memory storage occurs in two independent systems. The encoded information is temporarily stored in the hippocampus (36), the short-term memory storage center, and the short-term memory is continuously transferred to the neocortex, the long-term memory center, as time changes (37). When awake, the information encoded and stored in the cortex and hippocampus is constantly reproduced and activated in slow wave sleep and is shown as a dialog between the hippocampus and cortex on EEG. The cortical slow wave drives the hippocampus to the thalamus, generating spindle waves and sharp wave ripple/sharp wave ripple complex waves synchronously in a “top-down” manner (38). The synchronous appearance of spindle ripple makes the temporary memory in the hippocampus continuously transferred to the cortical memory network for consolidation and enhancement (39).

### 3.3. The arousal cycle affects synaptic change and cognition

Increased synaptic strength increases energy consumption and occupies more precious brain space, with fewer synaptic terminals during sleep and more synaptic terminals during wakefulness (40). Reducing the size of synaptic connections during sleep helps to save brain space and energy, thereby improving learning and memory. To ensure that synapses are not oversaturated and that neural signals and memories are not forgotten, studies have shown that this synaptic growth must be counteracted. Sleep is considered the perfect way to counteract this growth. During sleep, people can balance and restore synapses. A few hours of sleep reduced the synaptic size of the two cortical regions by 18%, and the synaptic resetting process was very ideal (41). Notably, the production of synaptic mRNA may only be regulated by the circadian rhythm of the biological clock, independent of the length and quality of sleep (42).

## 4. DORA improves sleep and alleviates cognitive impairment in AD

### 4.1. Hypnotics and cognition

There is widespread concern that hypnotic drugs may damage the body and cognitive functions, causing muscle relaxation, ataxia and loss of balance. Under the action of hypnotic drugs, patients need to stay awake and cannot make appropriate responses, resulting in falls or traffic accidents.

The launch of DORA will provide a new and important treatment option for insomnia patients. Compared with benzodiazepines and GABA drugs (43, 44), DORA could rapidly induce and maintain sleep by reducing orexin signaling, improve perceived sleep quality and have little impact on next-day waking performance. DORA did not affect posture stability or cognitive performance in the morning. Moreover, DORA slightly promoted cognition in animal experiments, allowing animals to perform well on the DSST scale and several attention tests, and seems to be expected to become an ideal hypnotic that does not impair cognition (45).

### 4.2. DORA reduces Aβ and tau deposition by improving sleep

Orexin physiologically promotes arousal by activating the monoaminergic system of arousal and inactivating the cholinergic network during REM sleep. Dysregulation of the orexin system leads to sleep/wakefulness disorder and then promotes **A**β deposition and tau-mediated neurodegeneration, thus accelerating cognitive decline in AD patients. Studies of APP/PS1 mice have demonstrated that DORA increases sleep, decreases **A**β plaque and tau protein formation in multiple brain regions, and indirectly affects biological clock function, such as circadian rhythm (46). Additionally, DORA mediates the long-term enhancement of the dentate gyrus of the hippocampus, which directly or indirectly alleviates cognitive impairment in AD by improving sleep activity (47).

## 5. Efficacy and safety of DORA in treating sleep/wakefulness disorders in patients with AD

### 5.1. DORA improves sleep

Plasma orexin-A levels are elevated in patients with chronic insomnia, resulting in difficulty sleeping and excessive brain awakening. Orexin neurons are active when awake and inactive during sleep (48). In theory, blocking orexin signals could induce insomnia patients to fall asleep and reduce nighttime wakefulness. Orexin neurons connect the ventrolateral preoptic area (which promotes sleep) and the brainstem (which promotes wakefulness), antagonizing cortical hyperexcitability and stabilizing the wake state by activating the arousal area and preventing unnecessary switching between wakefulness and sleep. Suvorexant, DORA, is a hypnotic drug developed by Mehr (70) that was approved by the US FDA in 2014. It can selectively inhibit the orexin signal-mediated arousal system to play a role in the transition of sleep (49).

### 5.2. DORA has fewer adverse reactions

When treating sleep problems in AD patients using antipsychotics and sedatives, potential adverse events in the deterioration of cognitive impairment have attracted attention (50). Several studies have shown that DORA is well tolerated in AD patients with insomnia, improving the total sleep time (TST) and reducing the number of night awakenings, and DORA does not seem to significantly change the basic sleep structural characteristics (51, 52), without evidence of potential cognitive impairment deterioration. As assessed by objective tests, DORA does not appear to impair cognitive or psychomotor performance on the following day.

DORA was previously found to improve the onset and maintenance of sleep after treatment at night for more than 3 months, which was generally safe and well tolerated. The most common related adverse events were mainly the expansion of pharmacological activity of the drug, namely, sleepiness, fatigue and dry mouth (53). The severity of lethargy is generally mild to moderate and rarely requires the discontinuation of treatment (54). Regarding the incidence of adverse reactions, the high-dose group reported more muscle weakness, strange dreams, sleepwalking, and abnormal psychological and behavioral symptoms than the low-dose group. Several normal attention processes of DORA subjects may also be affected, but whether the subjects should avoid driving the next day cannot be determined (55). DORA may also be beneficial to circadian rhythm disturbances, such as those among night-shift workers, who experience short daytime sleep time and poor quality, to restore the ideal sleep/wake-up time (56).

## 6. Conclusion

### 6.1. Intervention of the orexin system may be a potential target for AD

The cognitive performance of AD patients was negatively correlated with sleep latency and awakening time and positively correlated with sleep efficiency (57). The complex relationship between orexin and sleep/wakefulness disorder and the role of DORA in reducing Aβ deposition need to be clearly explained. The researchers hypothesized that a long-term loss of orexin early in life might alter Aβ metabolism, and the balance between production and degradation/clearance may prevent and delay the pathological process of AD (58).

However, the changes in sleep patterns from preclinical to clinical AD and the causal relationship with AD remain unclear. Few contradictory findings exist on the level of orexin in the CSF or plasma of AD patients (59). The neurobiological basis for explaining the relationship between sleep/wakefulness disorder and cognitive decline in AD remains to be elucidated. As a potential preventive treatment for AD, we hypothesize that DORA not only manages sleep/wakefulness disorders but also alleviates cognitive impairment by targeting the downregulation of the orexinergic system. Eventually, the neurodegenerative process of AD is slowed (60).

## 7. Prospect and expansion

### 7.1. Orexin system in other sleep disorders

In animal model discovery, dogs with hereditary narcolepsy symptoms were found to have abnormal orexin receptor-1 (OX1R) gene expression *in vivo*. In addition, mice with orexin gene deletion also showed sleep/wakefulness disorders, such as narcolepsy (61). In postmortem brain tissue samples from patients with narcolepsy, the number of orexin neurons were greatly reduced. Low CSF orexin-A levels are the gold standard (with high specificity and sensitivity) for the diagnosis of narcolepsy type 1 (62). People with narcolepsy have a lower risk of developing AD. However, the survey found that the incidence rate was similar to that of the general population. Does this result indicate that a normal amount of orexin is unnecessary in the pathogenesis of AD? Moreover, does this finding suggest that encouraging the use of orexin antagonists to delay the progression of AD is not warranted?

### 7.2. DORA and emotional disorders

Insomnia and depression are often mutually causal, with up to 40% of insomnia patients suffering from depression or anxiety (63). Animal models of stress and depression suggest that orexin-2 receptor antagonists have antidepressant activity (64). Human orexin-2 receptor antagonists counteract the effects of hyperexcitability by blocking inappropriate orexin release at night, thus alleviating depressive symptoms. Improving cognitive processing ability and central nervous system function is clearly beneficial. Specifically, a trend of subjective improvement in patients' mood was observed when taking medium and high doses of DORA (65). The findings indicate that the orexin system is involved in emotion regulation and antidepressant effects by improving sleep quality and sleep efficiency in patients with insomnia and depression.

### 7.3. Orexin receptors play an important role in drug addiction/reward

The role of orexin and its receptor in reward/addiction was first discovered in a behavioral study of conventional orexin knockout mice (66). Many studies have shown that the orexin system plays an important role in various seeking behaviors. Orexin 1 receptor (OX1R) antagonists have been proven to reduce the self-consumption of alcohol, nicotine or opioids. The hypothalamus is an important brain region in regulating natural reward (67). Orexin neurons are activated and mainly project to the paraventricular nucleus of the hypothalamus, ventral tegmental area (VTA) and nucleus accumbens, participating in emotional regulation disorder during withdrawal. Stronger withdrawal symptoms are related to lower levels of orexins (68, 69). Orexin projected to different brain regions has different regulatory effects on addiction caused by different drugs. The regulation of the orexin signaling system may become an important method in the treatment of addiction.

## Author contributions

MZ was responsible for writing and revising the article. ST was responsible for proposing ideas and conceiving the article. Both authors approved the submitted version.
